# Parent and Adolescent Reports of Adolescent Access to Household Firearms in the United States

**DOI:** 10.1001/jamanetworkopen.2021.0989

**Published:** 2021-03-09

**Authors:** Carmel Salhi, Deborah Azrael, Matthew Miller

**Affiliations:** 1 Bouvé College of Health Sciences, Department of Health Sciences, Northeastern University, Boston, Massachusetts; 2 Harvard Injury Control Research Center, Department of Health Policy and Management, T.H. Chan Harvard School of Public Health, Boston, Massachusetts

## Abstract

**Importance:**

Adolescent access to firearms increases their risk of firearm injury.

**Objective:**

To examine adolescent firearm access in homes with guns, how storage may modify access, and discordance between parent and adolescent report of access.

**Design, Setting, and Participants:**

This survey study used data on parents and their adolescent children from a nationally representative online survey of adults in gun-owning households, conducted from June 30 to August 11, 2019. Individuals who owned firearms and parents of adolescents were oversampled. All parents living with a child aged 13 to 17 years were invited to have their child participate in a separate survey. Respondents with more than 1 adolescent living with them were asked to choose the child with the most recent birthday. Analyses were conducted from June 1, 2020, to January 4, 2021.

**Main Outcomes and Measures:**

Firearm access was assessed by asking adolescents how long it would take to access a loaded gun in their home. Parents were asked whether their child could independently access a household firearm. Individuals who owned guns reported firearm storage practices. Study-specific poststratification weights adjusted for survey nonresponse and undercoverage or overcoverage resulting from the study-specific sample design and for benchmark demographic distributions.

**Results:**

Of 6721 adults invited to participate, 4030 completed the survey. Of these, 280 had a firearm in their homes and had a child aged 13 to 17 years who participated in the survey. The mean (SD) age of parents was 45.2 (7.2) years; of children, 15.0 (1.4) years. The sample included 159 male adolescents (weighted percentage, 60.8%; 95% CI, 53.8%-67.8%) and 129 male adults (weighted percentage, 48.3%; 95% CI, 40.9%-55.6%). In 33.9% (95% CI, 26.7%-41.2%) of households, an adolescent reported that they could access a loaded firearm in less than 5 minutes. In homes where all guns were locked, 23.7% (95% CI, 12.3%-35.1%) of adolescents reported that they could access a loaded firearm in less than 5 minutes. Overall, 70.4% (95% CI, 63.7%-77.1%) of parents reported that their child could not access a household firearm. In households where parents said their child could not access a firearm, 21.8% (95% CI 13.8%-29.7%) of their children indicated that they could access a firearm within 5 minutes and 14.9% (95% CI, 8.9%-20.9%) indicated that they could access a firearm in more than 5 minutes but less than 1 hour.

**Conclusions and Relevance:**

In this study, many adolescents reported having ready access to loaded guns in their homes, even when all household firearms were locked. Many adolescents who reported having access to household firearms lived with parents who knew their children had access, but others lived with parents who did not know. These finding should inform prevention efforts aimed at reducing adolescent access to household firearms.

## Key Points

**Question:**

Can adolescents in gun-owning homes access loaded firearms, and how often are parent’s reports of access discordant with their child’s?

**Findings:**

In this nationally representative survey study of 280 parent-child dyads who live in households with firearms, more than one-third of adolescents reported being able to access a loaded household firearm in less than 5 minutes; this proportion fell to nearly one-quarter when all firearms were locked. Although 70% of parents reported that their adolescent could not independently access a household firearm, more than one-third were contradicted by their child’s report.

**Meaning:**

Approaches to reducing firearm injury should focus on adolescents’ access to firearms, not solely on firearm storage.

## Teaser

This survey study examines adolescent firearm access in homes with guns, how storage may modify access, and discordance between parent and adolescent report of access.

## Introduction

The risk of suicide and unintentional firearm injury is several times higher for adolescents when they live in homes with firearms compared with children who lives in homes without them.^1,2,3,4,5^ For children and young adults in households with firearms, firearm injury is less likely when all firearms are stored locked and unloaded, as opposed to unlocked, loaded, or both unlocked and loaded.^2^ These observations underpin long-standing recommendations by the American Academy of Pediatrics for parents who choose to keep firearms in their home: lock up and unload all household firearms and store ammunition separately from the firearm.^6^

An issue that may get lost with a focus on in-home storage^7,8,9^ is the extent to which storing guns locked up, unloaded, or both effectively reduces youth access to loaded firearms in their home. While there is a large amount of literature on household firearm storage,^1,3,4,5,10,11^ the most recent of which estimates that approximately 10 million children live in households with at least 1 unlocked or loaded firearm,^12^ few studies have examined youth access to guns in their home.^12^ For example, the only nationally representative survey to have examined adolescent access to firearms in their homes found that between 2001 and 2004, 41% of youths aged 13 to 18 years who lived in homes with guns responded affirmatively to, “Could you get [to a household gun] and shoot it right now if you wanted to?”^13^ Two more recent studies^14,15^ among children presenting to emergency departments produced lower estimates. One found that 28% of adolescents aged 12 to 17 years reported being able to access a loaded firearm in less than 3 hours.^14^ The second found that 14% of children aged 7 to 17 years who lived in a household with firearms could “obtain a gun today.”^15^ The only data that speak to agreement between parent and child reports of access to firearms come from a study conducted in a family practice clinic in rural Alabama in 2002. In this study, approximately 1 in 5 parents who reported that their child (aged 5-14 years) had never handled a household gun were contradicted by their child’s report.^16^ To our knowledge, no study before ours has examined the association between access and firearm storage.

The current nationally representative survey describes adolescents’ access to household firearms in 2019 as reported by dyads of parents and their adolescent children. It extends prior work in 3 ways: (1) by describing contemporary youth access to household firearms, (2) by examining the association between firearm storage and youth firearm access, and (3) by reporting discordance in parent-child report of current access.

## Methods

### Sample and Data Collection

The present study uses data on parents and their adolescent children drawn from a nationally representative online survey (the 2019 National Firearms Survey [NFS]), conducted by the research firm Ipsos from July 30 to August 11, 2019. Respondents were drawn from Ipsos’s KnowledgePanel (KP), an online sampling frame comprising approximately 55 000 US adults recruited using address-based sampling methods. Samples selected from KP using probability-based recruitment methods are representative of the US population. Additional details can be found elsewhere.^17^ Eligible participants for the 2019 NFS were English-speaking noninstitutionalized US adults (18 years of age or older) residing in gun-owning households, excluding those on active duty in the US military. A gun-owning household was defined as a household where the respondent indicated either that they personally owned a gun or that someone else in their household did. Panel members’ reports of whether they lived in a home with firearms are collected on enrollment in KP and annually thereafter, allowing us to restrict invitations to adults who had previously reported that they lived in a home with firearms. Those who personally owned guns and parents or legal guardians of adolescents aged 13 to 17 years (hereafter referred to as parents) were oversampled.

Prior to asking parents any questions about firearms, any respondent who reported being the parent of and living with a child aged 13 to 17 years was invited to have their child participate in a separate survey. If the respondent had more than 1 child in that age group, they were asked to have the child with the most recent birthday participate. Reporting of response rates follows the Association for Public Opinion Research (AAPOR) definition for response rate 2, which includes partial interviews; partial interviews are defined as those who completed 50% to 80% of essential survey questions.^18^

Study-specific poststratification weights provided by Ipsos adjust for survey nonresponse and undercoverage or overcoverage resulting from the study-specific sample design. Poststratification weights also adjust for benchmark demographic distributions (from the US Census Current Population Survey [CPS] and the American Community Survey [ACS]) and for population characteristics, such as gun ownership (which are not available in the CPS or ACS), from weighted KP profile data.

The Harvard School of Public Health institutional review board approved the study. Panel members provide blanket consent to be recruited to participate in surveys on joining KP. Consent for this study was sought in the initial survey text seen by participants who could choose after reading the text to continue or not. These parents were also asked to allow their adolescent children to complete the survey in private and to let the adolescent know that they could decline to take the survey if they chose, skip any questions that make them uncomfortable, or stop taking the survey at any point.

### Adolescent-Reported Measures

Adolescents were asked whether “as far as you know, are there any guns in your home?” Adolescents who responded yes were asked additional questions about household firearms, including “How long would it take you to get 1 of the guns in your home (and load it, if it wasn't already loaded)?” Mutually exclusive response options were less than 5 minutes, less than 1 hour, less than 2 hours, and cannot get to it in less than 2 hours. Adolescents self-reported their age and gender. Adolescents’ age was dichotomized as 13 to 14 years or 15 to 17 years of age.

### Parent-Reported Measures

Parents who agreed to have their child participate were asked to complete an additional survey module that focused on the child who participated. To assess parent’s knowledge of the surveyed adolescent’s access to firearms, all parents were asked, “As far as you know, can your [surveyed] child access any of the guns in your home independently, that is without you or another adult in your house accessing it for him/her?” Possible responses were yes or no.

Respondents were asked whether they personally owned a gun. Gun-related information about the household (ie, number and type of guns, firearm storage) was elicited from parent respondents who personally owned guns to avoid measurement bias from adults who did not own guns, who have been known to underreport the presence of household firearms and overestimate how safely household firearms are stored.^19,20^ Individuals who owned guns were asked how many handguns they owned, how many long guns they owned, and how many guns in total were owned by other household members. Respondents who owned guns were also asked about the number of long guns and handguns they owned that were stored (1) loaded and unlocked, (2) unloaded and locked, (3) unloaded and unlocked, and (4) loaded and locked. Locking was defined as the use of a “trigger lock, cable lock, in a lock box or in another locked container.” For those who reported living with someone else who owned guns, they were also asked whether the other person who owned firearms stored any firearms (1) unlocked, (2) loaded, and (3) loaded and unlocked. These items were combined to identify whether the household had any firearms stored unlocked as well as whether the household had any firearms stored loaded. For households that had all firearms locked, we determined whether any locked guns were loaded. For households that had unlocked firearms, we determined whether any were loaded and unlocked.

### Statistical Analysis

Our analyses had 3 goals: (1) to describe the demographic and firearm-ownership characteristics of our sample of firearm-owning households with adolescents 13 to 17 years of age; (2) to estimate the proportion of adolescents who reported being able to gain access to household firearms in less than 5 minutes and in less than an hour and assess modifiers of reported access; and (3) to quantify the extent of discordance between parents’ and adolescents’ reports of adolescent access to firearms in the home. Discordance in reporting of adolescent access was noted when parents reported that their child could not access a firearm independently but the adolescent reported being able to access a gun in less than 5 minutes or less than 1 hour. Weighted percentages and their 95% CIs were estimated. Sampling weights supplied by Ipsos were applied such that all estimates are representative of households with firearms and children between 13 to 17 years of age. Description of sampling weights can be found in the eAppendix in the Supplement. Confidence intervals were set as 95% CIs (α = .05) using a 2-tailed distribution. Analyses were conducted from June 1, 2020, to January 4, 2021, in Stata version 16 (StataCorp).

## Results

Of the 6721 panel members invited to complete the survey regarding firearm ownership and storage-related behaviors, 4379 started and 4030 completed the survey (response rate, 65.2%; participation rate, 92.0%); 2950 of the 4030 (73.2%) personally owned firearms. Of the 408 survey respondents who indicated that they were the parent of a child between 13 to 17 years of age in their household, 318 participated with their child in the dyad survey (response rate 77.9%). Among the 318 adolescents who participated in the survey, 10 adolescents (3.1%) reported that there was no gun in their home, and 25 (7.9%) reported not knowing whether there was a gun in the home; these adolescents were not asked about access to firearms. Overall, 191 of 280 dyads (68.2%) included a parent who owned a firearm (89 parents lived in a household with firearms but did not personally own guns). The mean (SD) age of parents was 45.2 (7.2) years; of children, 15.0 (1.4) years. The sample included 159 male adolescents (weighted percentage, 60.8%; 95% CI, 53.8%-67.8%) and 129 male adults (weighted percentage, 48.3% [95% CI, 40.9%-55.6%]). Parents who participated in the dyad survey did not differ from those who did not participate in terms of gender, age, firearm ownership, or firearm storage.

### Descriptive Statistics of Firearm-Ownership Characteristics

Households where the parent respondent owned guns were similar to homes where the parent respondent did not personally own household guns, except that a larger proportion of parent respondents who owned guns were male compared with parents who did not own guns (123 parents [weighted percentage, 64.4%; 95% CI, 56.0%72.7%] vs 6 [weighted percentage, 8.9%; 95% CI, 0.4%-17.3%]) (Table 1). Parents who owned guns reported having a mean (SD) of 6.6 (8.1) firearms in the home; 84 (weighted percentage, 48.7%; 95% CI, 39.6%-57.7%) reported at least 1 household firearm was stored unlocked. Among households with any firearms unlocked, 43 (weighted percentage, 53.8%; 95% CI, 40.4%-67.2%) reported that at least 1 unlocked firearm was loaded (ie, 26.2% of all households had a loaded and unlocked gun) (Table 1).

**Table 1.** Demographic and Gun Ownership Descriptive Statistics of Sample^a^

Characteristic	No. (%) [95% CI]		
	Total (N = 280)	Gun ownership status	
		Yes (n = 191)	No (n = 89)
Age, mean (SD), y			
Adolescent	15.0 (1.4)	15.0 (1.4)	14.9 (1.5)
Parent	45.2 (7.2)	45.4 (7.5)	44.7 (6.5)
Household size, mean (SD), No.	4.3 (1.2)	4.2 (1.2)	4.6 (1.4)
Male participants			
Adolescent	159 (60.8) [53.8-67.8]	110 (63.5) [55.3-71.6]	49 (54.3) [41.2-67.3]
Parent	129 (48.3) [40.9-55.6]	123 (64.4) [56.0-72.7]	6 (8.9) [0.4-17.3]
Married or partnered parents	256 (92.8) [89.4-96.2]	173 (92.3) [88.3-96.3]	83 (93.9) [87.4-100]
Household guns, mean (SD), No.	NA	6.6 (8.1)	NA
Any guns stored			
Unlocked	NA	84 (48.7) [39.6-57.7]	NA
Loaded	NA	88 (50.4) [41.3-59.4]	NA
In homes with all guns locked, are any loaded	NA	38 (38.4) [26.9-50.0]	NA
In homes with any unlocked guns, are any loaded and unlocked	NA	43 (53.8) [40.4-67.2]	NA

Abbreviation: NA, not applicable.

^a^ All statistics weighted to be nationally representative of caregivers of adolescents 13 to 17 years of age in households with guns.

### Adolescent Report of their Access to Loaded Household Firearms 

One-third of adolescents reported that they could access a loaded firearm in under 5 minutes (33.9%; 95% CI, 26.7% 41.2%). An additional 17.4% (95% CI, 12.1% 22.6%) reported that they could gain access within an hour (Table 2).

**Table 2.** Adolescent Report of Time to Access a Household Firearm, by Gender, Age, and Household Gun Characteristics^a^

Characteristic	How long does it take to get to a loaded gun?							
	<5 min (n = 84)		5 min to <1 h (n = 52)		1 h to <2 h (n = 15)		Cannot access in <2 h (n = 129)	
	% (95% CI)	No. (weighted %)	% (95% CI)	No. (weighted %)	% (95% CI)	No. (weighted %)	% (95% CI)	No. (weighted %)
All adolescents	33.9 (26.7-41.2)	NA	17.4 (12.1-22.6)	NA	4.3 (1.7-6.8)	NA	44.4 (37.2-51.7)	NA
Parent’s ownership status								
Adult respondent does not own guns	36.4 (22.9-50.0)	27 (31.1)	18.4 (9.3-27.5)	18 (30.8)	4.9 (0.5-9.2)	6 (33.1)	40.3 (27.9-52.7)	38 (26.3)
Adult respondent does own guns	32.9 (24.5-41.4)	57 (68.9)	16.9 (10.5-23.4)	34 (69.3)	4.0 (0.9-7.2)	9 (66.9)	46.1 (37.3-54.9)	91 (73.7)
Adolescent gender								
Female	32.8 (21.3-44.3)	30 (37.7)	15.8 (8.4-23.3)	19 (36.2)	4.3 (0.8-7.8)	7 (39.3)	47.1 (36.2-57.9)	63 (41.5)
Male	34.9 (25.5-44.2)	54 (62.3)	18.0 (10.8-25.3)	32 (63.8)	4.3 (0.7-7.9)	8 (60.7)	42.8 (33.1-52.5)	65 (58.5)
Adolescent age, y								
13-14	29.7 (18.4-41.0)	27 (34.3)	13.8 (6.4-21.2)	17 (31.1)	2.0 (0.0-4.4)	4 (18.3)	54.5 (42.9-66.1)	63 (48.0)
15-17	36.7 (27.3-46.0)	57 (65.7)	19.7 (12.4-26.9)	35 (68.9)	5.7 (1.8-9.6)	11 (81.7)	38.0 (28.9-47.0)	66 (52.0)
Household guns, No.^b^								
1	18.8 (1.0-36.7)	5 (10.2)	11.5 (0.0-24.6)	4 (12.5)	6.1 (0.0-13.3)	3 (26.8)	63.6 (43.1-84.1)	21 (26.6)
2-4	30.4 (15.5-45.3)	16 (29.9)	21.8 (3.2-8.9)	14 (42.9)	4.4 (0.0-8.9)	4 (35.3)	43.4 (28.9-57.9)	31 (32.9)
≥5	43.0 (30.3-55.7)	36 (59.9)	16.0 (7.0-25.1)	14 (44.7)	3.3 (0.0-8.7)	2 (37.9)	37.6 (25.2-50.0)	36 (40.5)
Any guns stored unlocked^b^								
No	23.7 (12.3-35.1)	19 (39.4)	11.7 (3.7-19.7)	11 (39.0)	1.7 (0.0-3.7)	3 (20.7)	62.9 (50.6-75.1)	64 (73.6)
Yes	45.0 (31.8-58.2)	36 (60.6)	22.2 (11.3-33.1)	19 (61.0)	7.0 (0.5-13.4)	6 (79.3)	25.8 (14.0-37.6)	23 (26.4)
Any guns stored loaded^b^								
No	28.6 (15.4-41.8)	22 (41.8)	19.3 (8.8-29.8)	18 (57.1)	3.6 (0.3-6.9)	5 (41.8)	48.5 (35.0-62.0)	48 (53.5)
Yes	39.2 (27.6-50.9)	33 (58.2)	14.3 (5.8-22.8)	12 (42.9)	4.9 (0.0-10.7)	4 (58.2)	41.6 (29.9-53.2)	39 (46.5)
In homes with all guns locked, are any loaded^b^								
No	27.7 (11.6-43.7)	13 (68.6)	10.8 (0.7-20.9)	6 (52.0)	1.9 (0.0-4.5)	2 (66.8)	59.7 (43.1-76.2)	38 (56.5)
Yes	17.1 (3.8-30.5)	6 (31.4)	13.3 (0.2-26.3)	5 (48.0)	1.5 (0.0-4.5)	1 (33.2)	68.1 (51.1-85.1)	26 (43.5)
In homes with any unlocked guns, are any loaded and unlocked^b^								
No	28.3 (8.4-48.1)	10 (24.4)	32.0 (13.4-50.6)	14 (64.6)	6.7 (0.0-13.7)	4 (44.4)	33.0 (13.0-53.1)	13 (64.6)
Yes	59.4 (42.5-76.2)	26 (75.6)	13.8 (1.6-26.1)	5 (35.4)	7.2 (0.0-17.5)	2 (55.6)	19.6 (6.8-32.4)	10 (35.4)

Abbreviation: NA, not applicable.

^a^ All statistics weighted to be nationally representative of caregivers of adolescents 13 to 17 years of age in households with guns.

^b^ Statistics for number of guns in the home and firearm storage were reported by parent respondents who owned guns. Data were available for 191 parent-child dyads.

Nearly one-fifth of adolescents in homes with 1 firearm reported they could access a firearm in less than 5 minutes (18.8%; 95% CI, 1.0%-36.7%) compared with 43.0% (95% CI, 30.3%-55.7%) in households with 5 or more firearms. In households where all firearms were locked, nearly one-quarter of adolescents (23.7%; 95% CI, 12.3%-35.1%) reported being able to access a loaded firearm in less than 5 minutes and an additional 11.7% (95% CI, 3.7%-19.7%) within an hour. By contrast, in households with at least 1 unlocked firearm, 45.0% (95% CI, 31.8%-58.2%) of adolescents reported being able to access a loaded firearm in less than 5 minutes and an additional 22.2% (95% CI, 11.3%-33.1%) in less than an hour.

In households where all firearms were locked, having loaded guns did not substantially modify adolescents’ reports of their access to a loaded firearm. However, in households with unlocked firearms, the proportion of adolescents reporting being able to access a loaded firearm in less than 5 minutes was twice as high if at least 1 of the unlocked firearms was also stored loaded compared with a home where no unlocked guns were stored loaded (59.4% [95% CI, 42.5%-76.2%] vs 28.3% [95% CI, 8.4%-48.1%]).

### Agreement Between Parent and Adolescent Reports of Adolescent Access to Firearms 

Of the 70.4% (95% CI, 63.7%-77.1%) of parents who reported that their child could not access a firearm independently (80.3% [95% CI, 69.8%-90.8%] of parents who did not own guns and 66.3% [95% CI, 58.0%-74.7%] of parents who owned guns), 21.8% (95% CI, 13.8% 29.7%) of their adolescent children reported being able to access a loaded firearm within 5 minutes, and an additional 14.9% (95% CI, 8.9%-20.9%) reported being able to do so within an hour (Table 3). More parents who did not own guns than those who did were contradicted by their adolescent’s report of access, albeit with overlapping confidence intervals. For example, approximately 45% of parents who did not own guns and 33% of parents who owned guns and thought their adolescent could not access a household firearm had a child who reported they could, in fact, do so within an hour. Of the nearly one-third of parents who reported that their child could access a firearm independently (19.7% [95% CI, 9.2%-30.2%] of parents who did not own guns and 33.7% [95% CI, 25.3%-42.0%] of parents who owned guns), most were corroborated by their child’s report (nearly two-thirds within 5 minutes and more than 85% within 1 hour).

**Table 3.** Adolescent Report of Firearm Access by Parent Report of Access^a^

Adolescent report	% (95% CI)					
	Parents report that their child can gain access to a household gun^b^		Parents who did not own a gun reported that their child can gain access to a household gun^b^		Parents who own a gun reported that their child can gain access to a household gun^b^	
	No (n = 197)	Yes (n = 82)	No (n = 69)	Yes (n = 20)	No (n = 128)	Yes (n = 62)
Parent's report of adolescent access, overall	70.4 (63.7-77.1)	29.6 (22.9-36.3)	80.3 (69.8-90.8)	19.7 (9.2-30.2)	66.3 (58.0-74.7)	33.7 (25.3-42.0)
Adolescent report of how long to get a gun and load it						
<5 min	21.8 (13.8-29.7)	63.3 (50.4-76.2)	26.8 (11.8-41.8)	75.8 (53.1-98.4)	19.3 (10.2-28.4)	60.3 (45.5-75.1)
5 min to <1 h	14.9 (8.9-20.9)	22.2 (11.6-32.7)	18.0 (8.1-28.0)	19.8 (0.0-41.5)	13.4 (5.8-20.9)	22.7 (10.7-34.8)
1 h to <2 h	4.6 (1.9-7.3)	3.6 (0.0-9.5)	6.1 (0.7-11.5)	0.0 (NA)	3.8 (0.9-6.8)	4.5 (0.0-11.7)
Can't access in <2 h	58.7 (50.0-67.4)	10.9 (2.7-19.2)	49.1 (34.6-63.5)	4.4 (0.0-10.9)	63.5 (52.9-74.1)	12.5 (2.4-22.6)

^a^ All statistics weighted to be nationally representative of caregivers of adolescents 13 to 17 years of age in households with guns.

^b^ Based on parent question, “As far as you know, can your child access any of the guns in your home independently, that is without you or another adult in your house accessing it for him/her?”

## Discussion

Four key findings emerged from our analysis. First, in at least one-third of gun-owning households with an adolescent 13 to 17 years of age, adolescents reported that they could access a loaded firearm in their home in less than 5 minutes (half reported being able to do so within an hour). Second, while unlocked firearm storage was associated with greater adolescent access, in homes where all guns were locked 1 in 4 adolescents reported being able to access a loaded firearm in less than 5 minutes (and 1 in 3 in under an hour). Third, in more than one-quarter of gun-owning households, parents reported that their adolescent child could independently gain access to a household firearm. And fourth, among the 70% of households where parents did not believe their child could access a household firearm, more than 1 in 5 adolescents reported being able to access a loaded household firearm within 5 minutes (37% within an hour).

To our knowledge, no prior nationally representative study has examined the ways in which household firearm storage practices are associated with adolescent access to firearms. Our finding that storage practices are imperfect proxies for adolescent access suggests that the advice to lock all household firearms, as recommended by several professional medical associations, should always incorporate the caveat, now empirically grounded, that locking all firearms does not necessarily prevent access. In addition, our discordance findings suggest that efforts to reduce adolescent access to firearms in households may benefit from engaging parents with their adolescents in conversations about access to household firearms, especially when parents think their child cannot access a household gun. These conversations might include efforts to make the parent who does not personally own a firearm aware that they are especially likely to misjudge their child’s independent access to a gun in the home. Finally, our finding that 1 in 4 parents said that their adolescent can independently access household firearms suggests that a nontrivial proportion of parents view such access as likely a net benefit (or at least not a net risk). If so, this finding suggests that millions of US parents underestimate the injury risk or overestimate the protective benefit that ready access confers on their adolescents.

### Limitations

Five aspects of our study design should be considering when interpreting our results. First, although our sample of adolescent-parent dyads is the largest of which we are aware in the published peer-reviewed literature, our sample size limits the precision of estimates. In 64% of gun-owning parent–adolescent dyads, the adolescent was male (54% of non–gun-owning dyads were male). This imbalance may have been due to chance alone (the confidence intervals largely overlap). It is also possible that parents who own guns preferentially called on their male children to answer the survey. Because male and female adolescents do not differ materially in their report of access to household guns (or of parent-child agreement regarding access; data not shown), poststratification weighting by adolescent gender would not meaningfully change our point estimates. Second, some adolescents responded that they either did not know if or did not think there was a gun in the home. If we assigned these adolescents to not being able to access a firearm in under 2 hours, our overall estimates of adolescent access to a loaded household firearm would be slightly, but not significantly, lower than reported in the Results section: 30.2% (95% CI, 23.6%-36.8%) would have reported that they could access a gun in less than 5 minutes and an additional 15.4% (95% CI, 10.7%-20.2%) within an hour. Third, we surveyed only 1 adolescent per household. It is possible that in households where the adolescent respondent reported no firearm access, another adolescent had access. Accordingly, our results are a lower bound estimate of the number of households that have at least 1 adolescent who can access a firearm. Fourth, we asked children how quickly they could gain access to a loaded household gun but did not further specify (eg, we did not indicate that the question pertained to independently gaining access without seeking assistance from an adult). Fifth, while we asked parents whether they knew if their child could access a household firearm, we did not ask how parents felt about their child having access to guns in their home; whether, and in what ways, parents may have discouraged or encouraged access; or under what conditions parents would be willing to take additional precautions to reduce access. Furthermore, because parents were asked about their adolescent’s access to household firearms in general (ie, not access to loaded firearms), whereas adolescents were asked about access to a loaded household gun, some parents may have reported that their child had access to a household gun when they would have reported otherwise had they been asked about access to a loaded gun. Because of this, the discordance we reported with respect to access to a household gun is a lower bound compared with what we might have found had we asked parents about their child’s access to loaded firearms.

## Conclusions

Prevention efforts aimed at reducing access to household firearms should be informed by the findings of this study. They were that (1) many adolescents reported having ready access to loaded guns in their home, even when all household firearms are locked, (2) many adolescents who reported having access to household firearms lived with parents who knew they had access, and (3) many others live with parents who did not.

## Notes

Supplement. **eAppendix.** Description of Sample and Survey Weights

## References

[1] Miller M, Hemenway D. The relationship between firearms and suicide: a review of the literature. Aggress Violent Behav. 1999;4(1):59–75. doi:10.1016/S1359-1789(97)00057-8

[2] Grossman DC, Mueller BA, Riedy C, . Gun storage practices and risk of youth suicide and unintentional firearm injuries. JAMA. 2005;293(6):707-714. doi:10.1001/jama.293.6.707 15701912

[3] Brent DA, Perper JA, Moritz G, Baugher M, Schweers J, Roth C. Firearms and adolescent suicide: a community case-control study. Am J Dis Child. 1993;147(10):1066-1071. doi:10.1001/archpedi.1993.02160340052013 8213677

[4] Brent DA, Perper JA, Allman CJ, Moritz GM, Wartella ME, Zelenak JP. The presence and accessibility of firearms in the homes of adolescent suicides: a case-control study. JAMA. 1991;266(21):2989-2995. doi:10.1001/jama.1991.03470210057032 1820470

[5] Anglemyer A, Horvath T, Rutherford G. The accessibility of firearms and risk for suicide and homicide victimization among household members: a systematic review and meta-analysis. Ann Intern Med. 2014;160(2):101-110. doi:10.7326/M13-1301 24592495

[6] Dowd MD, Sege RD; Council on Injury, Violence, and Poison Prevention Executive Committee; American Academy of Pediatrics. Firearm-related injuries affecting the pediatric population. Pediatrics. 2012;130(5):e1416-e1423. doi:10.1542/peds.2012-2481 23080412

[7] Rowhani-Rahbar A, Simonetti JA, Rivara FP. Effectiveness of interventions to promote safe firearm storage. Epidemiol Rev. 2016;38(1):111-124. doi:10.1093/epirev/mxv006 26769724

[8] Albright TL, Burge SK. Improving firearm storage habits: impact of brief office counseling by family physicians. J Am Board Fam Pract. 2003;16(1):40-46. doi:10.3122/jabfm.16.1.40 12583649

[9] Miller M, Salhi C, Barber C, . Changes in firearm and medication storage practices in homes of youths at risk for suicide: results of the SAFETY Study, a clustered, emergency department-based, multisite, stepped-wedge trial. Ann Emerg Med. 2020;76(2):194-205. doi:10.1016/j.annemergmed.2020.02.007 32307124

[10] Miller M, Azrael D, Hemenway D. Firearm availability and unintentional firearm deaths, suicide, and homicide among 5-14 year olds. J Trauma. 2002;52(2):267-274. doi:10.1097/00005373-200202000-00011 11834986

[11] Miller M, Azrael D, Hemenway D, Vriniotis M. Firearm storage practices and rates of unintentional firearm deaths in the United States. Accid Anal Prev. 2005;37(4):661-667. doi:10.1016/j.aap.2005.02.003 15949457

[12] Azrael D, Cohen J, Salhi C, Miller M. Firearm storage in gun-owning households with children: results of a 2015 national survey. J Urban Health. 2018;95(3):295-304. doi:10.1007/s11524-018-0261-7 29748766 PMC5993703

[13] Simonetti JA, Mackelprang JL, Rowhani-Rahbar A, Zatzick D, Rivara FP. Psychiatric comorbidity, suicidality, and in-home firearm access among a nationally representative sample of adolescents. JAMA Psychiatry. 2015;72(2):152-159. doi:10.1001/jamapsychiatry.2014.1760 25548879

[14] Pelucio M, Roe G, Fiechtl J, . Assessing survey methods and firearm exposure among adolescent emergency department patients. Pediatr Emerg Care. 2011;27(6):500-506. doi:10.1097/PEC.0b013e31821d8643 21629145

[15] Doh KF, Morris CR, Akbar T, . The relationship between parents’ reported storage of firearms and their children’s perceived access to firearms: a safety disconnect. Clin Pediatr (Phila). 2021;60(1):42-49. doi:10.1177/0009922820944398 32748645

[16] Baxley F, Miller M. Parental misperceptions about children and firearms. Arch Pediatr Adolesc Med. 2006;160(5):542-547. doi:10.1001/archpedi.160.5.542 16651499

[17] Salhi C, Azrael D, Miller M. Patterns of gun owner beliefs about firearm risk in relation to firearm storage: a latent class analysis using the 2019 National Firearms Survey. Inj Prev. 2020;injuryprev-2019-043624. doi:10.1136/injuryprev-2019-043624 32665253

[18] Smith TW. Standard Definitions: Final Dispositions of Case Codes and Outcome Rates for Surveys. AAPOR; 2015. Accessed February 2, 2021. https://www.aapor.org/AAPOR_Main/media/publications/Standard-Definitions20169theditionfinal.pdf

[19] Ludwig J, Cook PJ, Smith TW. The gender gap in reporting household gun ownership. Am J Public Health. 1998;88(11):1715-1718. doi:10.2105/AJPH.88.11.1715 9807545 PMC1508567

[20] Azrael D, Miller M, Hemenway D. Are household firearms stored safely? it depends on whom you ask. Pediatrics. 2000;106(3):E31. doi:10.1542/peds.106.3.e31 10969115

