# Supplementary material for: Parent and Adolescent Reports of Adolescent Access to Household Firearms in the United States
Source: JAMA Netw Open. 2021 Mar 9;4(3):e210989. doi: 10.1001/jamanetworkopen.2021.0989 (PMC7944379; doi:10.1001/jamanetworkopen.2021.0989)
Supplement: Supplement. — eAppendix. Description of Sample and Survey Weights [file jamanetwopen-e210989-s001.pdf]

Supplemental Online Content

Salhi C, Azrael D, Miller M. Parent and adolescent reports of adolescent access to household firearms in the United States. *JAMA Netw Open*. 2021;4(3):e210989. doi:10.1001/jamanetworkopen.2021.0989

eAppendix. Description of Sample and Survey Weights

This supplemental material has been provided by the authors to give readers additional information about their work.

© 2021 Salhi C et al. *JAMA Network Open.*

eAppendix. Description of Sample and Survey Weights

Ipsos uses an address-based recruitment process for KnowledgePanel such that active panel members are representative the adult population of the U.S. Panel members are recruited using an addressed-based sampling methodology that uses the latest Delivery Sequence File of the USPS – a database with full coverage of all delivery points in the US. For selection of general population samples from KnowledgePanel, Ipsos weights these samples to behave as equal probability selection samples. Briefly, this methodology starts by weighting the pool of active members to the geodemographic benchmarks secured from the latest March supplement of the U.S. Census Bureau's Current Population Survey (CPS) by gender, age, race and Hispanic ethnicity, education, census region, household income, home ownership status and metropolitan area. Using the resulting weights as measures of size, a probability-proportional-to-size (PPS) procedure is used to select study specific samples. The application of PPS methodology with the imposed size measures produces fully self-weighing samples from KnowledgePanel, for which each sample member can carry a design weight of unity. Oversampling of subgroups is accounted for by adjusting the design weights in reference to the CPS benchmarks for the population of interest.

Once the study sample has been selected and fielded, and all the survey data are edited and made final, design weights are adjusted for any survey nonresponse as well as any under- or over-coverage imposed by the study specific sample design. Depending on the specific target population for a given study, geodemographic distributions for the corresponding population are obtained from the CPS, the American Community Survey (ACS), or in certain instances from the weighted KP profile data. For weighting adjustments, an iterative proportional fitting (raking) procedure is used to produce final weights that will be aligned with respect to all study

benchmark distributions simultaneously. In the final step, calculated weights are examined to identify and, if necessary, trim outliers at the extreme upper and lower tails of the weight distribution. The resulting weights are then scaled to the sum of the total sample size of all eligible respondents.

© 2021 Salhi C et al. *JAMA Network Open.*
